# The Use of Nitro-glycerine

**Published:** 1887-01

**Authors:** 


THE use of nitroglycerine by physicians should be attended with some special care. The drug may be old and inert, or, on the other hand, the alcohol which holds it in solution may evaporate and leave the solution dangerously strong.—*Medical Record.*

[Consistent homœopathists do not need this caution.—EDS.]

